# Ovarian hemangioma occurring synchronously with contralateral mature cystic teratoma in an 81-year-old patient

**DOI:** 10.3109/03009734.2010.502602

**Published:** 2010-10-27

**Authors:** Cem Comunoglu, Latife Atasoy, Cem Baykal

**Affiliations:** ^1^Başkent University, Faculty of Medicine, Istanbul Hospital, Department of Pathology, Altunizade, Uskudar, IstanbulTurkey; ^2^Başkent University, Faculty of Medicine, Istanbul Hospital, Department of Gynecology and Obstetrics, Altunizade, Uskudar, IstanbulTurkey

**Keywords:** Cavernous hemangioma, mature cystic teratoma, ovarian hemangioma, ovarian neoplasm

## Abstract

**Purpose:**

Ovarian hemangiomas are seen rarely. We present a case of an ovarian hemangioma occurring synchronously with contralateral mature cystic teratoma.

**Case history:**

An 81-year-old woman presented with hypertension and hyponatremia. In ultrasonographic evaluation a pelvic mass was found located at the left ovary. Histologically, a mature cystic teratoma measuring 9.5 × 9 × 8 cm was seen in left ovary. In the right ovary an incidental vascular lesion measuring 3.5 × 1.5 × 1 cm was observed. Final histopathological examination of this lesion demonstrated a hemangioma of cavernous type.

**Conclusion:**

To the best of our knowledge, this is the first ovarian hemangioma case occurring synchronously with contralateral mature cystic teratoma.

## Introduction

Hemangiomas of the ovary are very rare tumors mostly seen incidentally at operation or autopsy. They sometimes coexist with genital tract diseases even with malignancies (1). It has been proposed that hemangiomas originate from germ cells as part of a teratoma (2). We will present a rare ovarian hemangioma occurring synchronously with contralateral mature cystic teratoma enabling us to observe and evaluate the hemangioma case in terms of vascular components and compare it with a teratoma.

## Case report

### Clinical findings

An 81-year-old female patient was admitted to the nephrology department with hypertension and hyponatremia. Clinical history did not reveal vaginal bleeding or any other gynecologic complaint. In ultrasonographic evaluation of the renal system, a pelvic mass was found located at the left ovary. A detailed abdominal ultrasonography and computed tomography (CT) scan showed a left ovarian mass. Tumor markers were within the normal range (Ca-125: 4.64 U/mL; Ca-15.3: 5.4 U/mL; Ca-19.9: 0 U/mL; CEA: 1.3 ng/mL), and there was no ascites.

During laparotomy, a cystic mass located at the left ovary was found. Uterus and the right ovary appeared normal. She underwent total hysterectomy and bilateral salpingo-oophorectomy. The left ovarian cystic lesion was sent to intraoperative frozen consultation and was reported as a mature cystic teratoma.

### Pathological findings

#### Macroscopic findings

Left ovary measured 9.5 × 9 × 8 cm, and the cyst wall was 0.1 cm in thickness. Right ovary seen attached to the uterus measured 4 × 2.5 × 1.5 cm. On cut section an incidental vascular lesion measuring 3.5 × 1.5 × 1 cm was observed. The lesion was relatively distinct from the adjacent ovarian stroma. The uterus was unremarkable.

#### Histopathological findings

**Figure 1.**
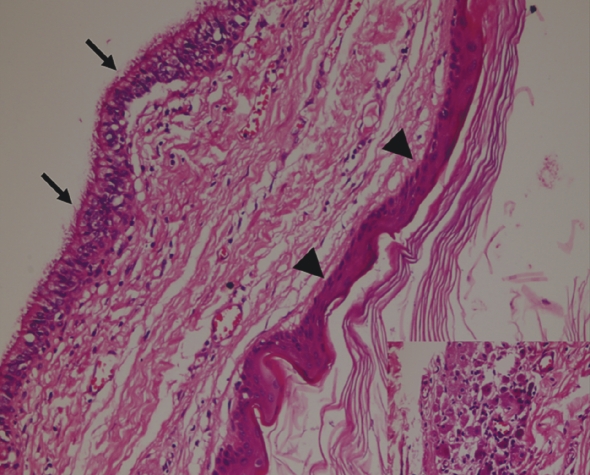
Left ovarian cyst, lined by keratinous squamous epithelium (arrow-heads) and cystic formation lined by ciliated columnar epithelium in the subepithelial region (arrows) (Hematoxylin and Eosine, ×100). Inset: Mature ganglion cells (Hematoxylin and Eosine, ×200).

Microscopically, the left ovarian cyst was lined by keratinous squamous epithelium. In the subepithelial region, mature adipous tissue, sero-mucous glands, mature ganglion cells, and peripheral nerve tissue were observed (Figure 1). Malignant or immature components were not noted.

**Figure 2.**
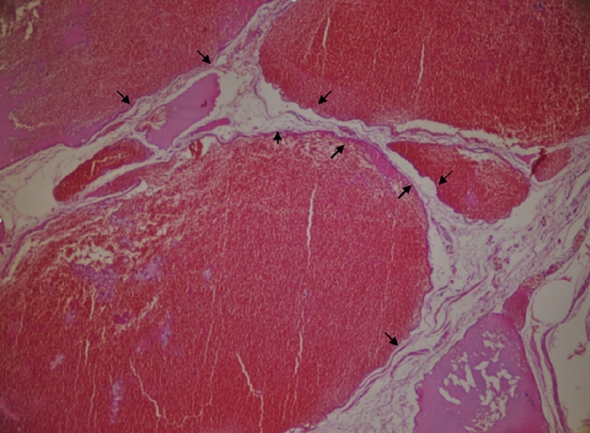
Dilated thin-walled vessels (arrows) lined by a single layer of endothelial cells, containing red blood cells in their lumen (Hematoxylin and Eosine, ×40).

Sections of right ovarian vascular lesion revealed dilated thin-walled vessels, containing red blood cells in their lumen, lined by a single layer of endothelial cells (Figure 2). Teratomatous components were not detected in surrounding stroma. A diagnosis of a hemangioma of cavernous type was made.

## Discussion

Hemangiomas of the ovary are seen very rarely. Most ovarian hemangiomas are of the cavernous type and are found incidentally after operation or autopsy. They may coexist with different genital tract diseases (3), but to the best of our knowledge this is the first case report of an ovarian cavernous hemangioma synchronous with contralateral mature cystic teratoma.

The origin of ovarian hemangioma is controversial. It has been suggested that hemangiomas are part of a mature teratoma, or benign true ovarian neoplasms, or hamartomatous malformations (2).

Hemangioma must be differentiated from proliferations of dilated blood vessels of the ovarian hilar region (4). It has been proposed that a circumscribed nodule or a mass tended to distinguish hemangiomas from hilar vascular proliferations (5). In our case we have observed a relatively well demarcated mass lesion consisting of dilated vessels with little stroma, indicating a diagnosis of hemangioma.

In differential diagnosis a mature cystic teratoma should be distinguished from a hemangioma. Mature cystic teratoma of the ovary with florid vascular proliferation has been reported (6). In our case the matter is very intriguing because of the presence of a mature cystic teratoma of the contralateral ovary. It has been reported that 8%–15% of cases are bilateral (7). It is specifically recommended for ovarian hemangiomas that in order to exclude a teratoma, teratomatous components should be diligently searched for with extensive sampling. For the present case this was especially important because of a possibility of a bilateral teratoma. In our case we could not detect any teratomatous components in the right ovary. Extensive sampling of the mature cystic teratoma of the left ovary did not reveal any prominent vascular component. These findings suggest that these two tumors coexist independently and do not support the hypothesis of hemangiomas originating from germ cells.

In conclusion, we here present an ovarian hemangioma case occurring synchronously with contralateral mature cystic teratoma, to the best of our knowledge the first in the English literature.

## References

[1] Gucer F, Ozyilmaz F, Balkanli-Kaplan P, Mulayim N, Aydin O (2004). Ovarian hemangioma presenting with hyperandrogenism and endometrial cancer: a case report. Gynecol Oncol.

[2] Itoh H, Wada T, Michikata K, Sato Y, Seguchi T, Akiyama Y (2004). Ovarian teratoma showing a predominant hemangiomatous element with stromal luteinization: report of a case and review of the literature. Pathol Int.

[3] Akbulut M, Bir F, Çolakoğlu N, Soysal ME, Düzcan SE (2008). Ovarian hemangioma occurring synchronously with serous papillary carcinoma of the ovary and benign endometrial polyp. Ann Saudi Med.

[4] Uppal S, Heller DS, Majmudar B (2004). Ovarian hemangioma-report of three cases and review of the literature. Arch Gynecol Obstet.

[5] Talerman A, Kurman RJ (2002). Nonspecific tumors of the ovary, including mesenchymal tumors and malignant lymphoma. Blaustein's Pathology of the Female Genital Tract.

[6] Akbulut M, Zekioglu O, Terek MC, Ozdemir N (2009). Florid vascular proliferation in mature cystic teratoma of the ovary: case report and review of the literature. Tumori.

[7] Peterson WF, Prevost EC, Edmunds FT, Hundley JM, Morris FK (1955). Benign cystic teratomas of the ovary; a clinico-statistical study of 1,007 cases with a review of the literature. Am J Obstet Gynecol.

